# Experimental Evidence for a Revision in the Annotation of Putative Pyridoxamine 5'-Phosphate Oxidases P(N/M)P from Fungi

**DOI:** 10.1371/journal.pone.0136761

**Published:** 2015-09-01

**Authors:** Tatiana Domitrovic, Diana P. Raymundo, Tiago Fernandes da Silva, Fernando L. Palhano

**Affiliations:** 1 Departamento de Virologia, Instituto de Microbiologia Paulo de Goes, Universidade Federal do Rio de Janeiro, Rio de Janeiro, Brazil; 2 Programa de Biologia Estrutural, Instituto de Bioquímica Médica Leopoldo de Meis, Universidade Federal do Rio de Janeiro, Rio de Janeiro, Brazil; 3 Laboratório de Avaliação e Síntese de Substâncias Bioativas (LASSBio), Instituto de Ciências Biomédicas, Universidade Federal do Rio de Janeiro, Rio de Janeiro, Brazil; 4 Programa de Graduação em Química (PGQu), Instituto de Química, Universidade Federal do Rio de Janeiro, Rio de Janeiro, Brazil; National Research Council of Italy (CNR), ITALY

## Abstract

Pyridoxinamine 5'-phosphate oxidases (P(N/M)P oxidases) that bind flavin mononucleotide (FMN) and oxidize pyridoxine 5'-phosphate or pyridoxamine 5'-phosphate to form pyridoxal 5'-phosphate (PLP) are an important class of enzymes that play a central role in cell metabolism. Failure to generate an adequate supply of PLP is very detrimental to most organisms and is often clinically manifested as a neurological disorder in mammals. In this study, we analyzed the function of *YLR456W* and *YPR172W*, two homologous genes of unknown function from *S*. *cerevisiae* that have been annotated as putative P(N/M)P oxidases based on sequence homology. Different experimental approaches indicated that neither protein catalyzes PLP formation nor binds FMN. On the other hand, our analysis confirmed the enzymatic activity of Pdx3, the *S*. *cerevisiae* protein previously implicated in PLP biosynthesis by genetic and structural characterization. After a careful sequence analysis comparing the putative and confirmed P(N/M)P oxidases, we found that the protein domain (PF01243) that led to the *YLR456W* and *YPR172W* annotation is a poor indicator of P(N/M)P oxidase activity. We suggest that a combination of two Pfam domains (PF01243 and PF10590) present in Pdx3 and other confirmed P(N/M)P oxidases would be a stronger predictor of this molecular function. This work exemplifies the importance of experimental validation to rectify genome annotation and proposes a revision in the annotation of at least 400 sequences from a wide variety of fungal species that are homologous to *YLR456W* and are currently misrepresented as putative P(N/M)P oxidases.

## Introduction

Knowledge of the functions of the proteins within an organism is key for understanding life at the molecular level. However, due to the inherent complexity of this challenge, the experimental characterization of protein function evolves much more slowly than does the number of deposited sequences from many organisms [1,2].

Most genome databases organize their functional information according to Gene Ontology (GO). GO is a standardized, structured vocabulary that is used to annotate gene products according to the molecular functions that they perform, the biological processes in which they participate, and the cellular components with which they are associated. GO annotations can be based on experimental data, i.e., curated from the scientific literature available for each particular gene product. This annotation process usually requires PhD-level scientific curators who select and prioritize the relevant information to generate a coherent GO annotation that reflects the current understanding about a gene product. Though this process is considered the gold standard for genome annotation, many databases have been utilizing automated annotation methods based on sequence comparison. This type of annotation is faster and uniform across all gene products, can be easily updated and does not require any experimental work [3].

The *Saccharomyces* genome database (SGD, http://www.yeastgenome.org) is the community resource for the yeast *Saccharomyces cerevisiae* and manages the associated information for the best-annotated genome of all of the eukaryotic organisms [4]. SGD compiles manually curated information from the peer-reviewed literature and provides computationally based annotations that have not been reviewed by a curator and are directly extracted from the Gene Ontology Annotation (GOA) project of the European Bioinformatics Institute (EBI). These computational annotations are predictions that can guide the functional assignment of gene products that have unknown functions. Most of the predictions are based on protein sequence classification methods. GOA uses InterPro and Pfam entries, which represent conserved protein sequence patterns such as domains, motifs, active sites or protein family signatures that are then associated with GO terms denoting the function of proteins containing a particular pattern [5,6].

The computational annotation of gene products has already proved its value to guide laboratory experiments and improve gene annotation. In an elegant work, Hibbs and colleagues showed a 238% increase in the discovery rate for computationally selected genes over randomly selected genes when searching for the proteins implicated in mitochondrial biogenesis [7]. However, the ability of the automated methods to accurately predict protein function is still limited. The critical assessment of protein function annotation (CAFA) project [8,9] was designed to measure the accuracy of the computational annotation methods. Fifty-four methods were used to predict the function of 50000 unknown proteins of several different organisms. Eleven months after the submission deadline, 866 of those proteins were experimentally annotated and became the reference set for evaluating the efficiency of the predictions. The performance of different prediction methods (e.g. BLAST) was calculated by the maximum F measure (Fmax), which considers predictions across the full spectrum from high to low sensitivity. In this metric, a perfect prediction method would be characterized with Fmax = 1. The actual performances for the molecular function predictions ranged from 0.6 to 0.4, for the best and the worst prediction methods, respectively. Although CAFA metrics have been debated, the consensus is that there is a considerable need for improved computational predictions and that the characterization of gene functions must be carefully validated by other approaches such as classical genetics, biochemistry or structural biology [8].

**Fig 1:**
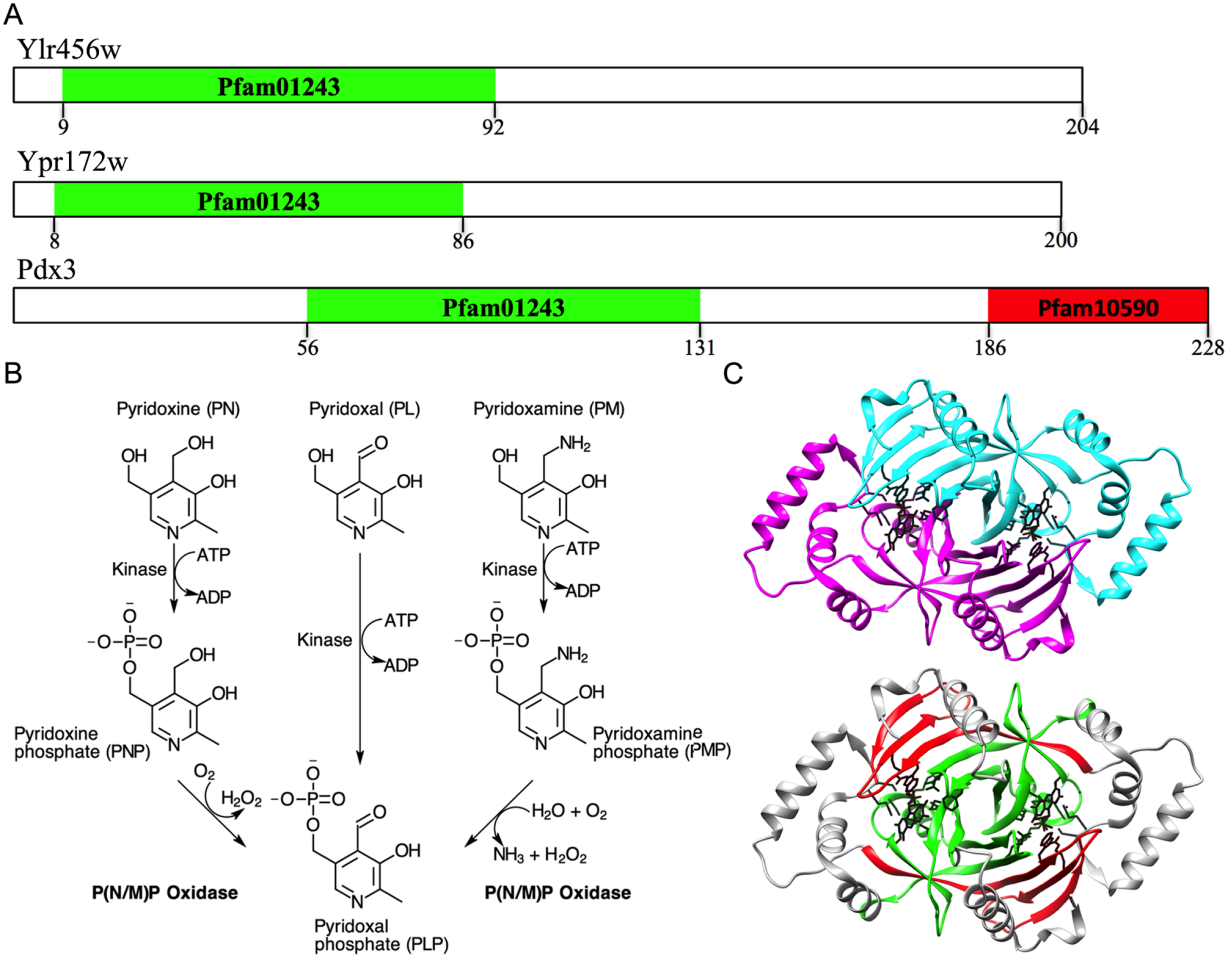
Ylr456w and Ypr172w annotations are inferred from only one domain involved in pyridoxamine 5’-phosphate function. (A) Pfam protein family database profile of the proteins Ylr456w, Ypr172w and Pdx3. PF01243 Pyridox_oxidase domain and PF10590 Pyridox_oxidase C-terminal dimerization domain are located on the N-terminal and C-terminal, respectively. (B) Vitamin B6 biosynthesis. (C) Crystal structure of the P(N/M)P oxidase Pdx3 (pdb 1CI0) from *S*. *cerevisiae*. The P(N/M)P oxidase protein is a homodimer (monomers colored in cyan and magenta) that binds two FMN molecules. The side chain of the conserved residues that contact FMN and their ligands are shown in black (residues R73, L76, K96, Q153, S154, E197, W199, R205, H207, R209 and P228 are indicated by red asterisks in Fig 4). The second structure (bottom) was colored to show the tridimensional position of the amino-acid residues within PF01243 in green and PF10590 in red.

In this study, we used different experimental approaches to verify the annotation of two unknown yeast genes, *YLR456W* and its homolog *YPR172W*. These two genes are closely related and were originated by genome duplication [10]. Both are computationally assigned to the GO term “pyridoxamine phosphate oxidase” in SGD (Fig 1A, Pfam01243). This class of enzymes is involved in vitamin B6 metabolism and plays a central role in amino-acid biosynthesis and other important pathways [11]. Vitamin B6 is a generic term for pyridoxine (PN), pyridoxal (PL), pyridoxamine (PM) and their related phosphorylated forms (PNP, PLP and PMP, respectively). Pyridoxamine phosphate oxidases are enzymes that convert PMP or PNP into PLP (Fig 1B), and most PNP oxidases possess PMP oxidase activity as well, so they are known as P(N/M)P oxidases (Fig 1B). PLP is the most versatile organic cofactor in biology, and production deficiencies in mammals are often clinically manifested as neurological disorders [11]. All P(N/M)P oxidases form stable dimers that contain an FMN-binding split barrel fold and require flavin mononucleotide (FMN) to be active [12].

In addition to *YLR456W* and *YPR172W*, there is just one more entry in SGD for the GO term “pyridoxamine phosphate oxidase”: the gene *PDX3*, which was experimentally characterized as the yeast P(N/M)P oxidase three decades ago by Loubbardi et al. The characterization was based on enzymatic activity of crude yeast extracts of WT and *PDX3* deleted strains [13]. Later, the *PDX3* gene was cloned, and the protein structure elucidated (pdb 1CI0), which revealed a classic P(N/M)P oxidase fold, typical of the flavin-containing oxidase that is conserved from *E*. *coli* to humans: a compact eight-stranded β-sheet core that is surrounded by five α-helical structures. The two monomers create two enclosed cavities, each containing a bound FMN molecule (Fig 1C) [12].

Ylr456w and Ypr172w are conserved across a wide range of fungal species but are quite different from Pdx3. The homology is based solely on the conservation of a short sequence in the N-terminal domain that is characteristic of P(N/M)P oxidases (Fig 1A, Pfam01243). Given the potential involvement of these two unknown ORFs in such an important metabolic role, we conducted experiments to test whether *YLR456W* and *YPR172W* gene products function as a P(N/M)P oxidase. We found that the recombinant Ylr456w and Ypr172w proteins form homodimers that cannot bind FMN and do not possess P(N/M)P oxidase activity. On the other hand, Pdx3 recombinant protein exhibits all expected properties of a bona fide P(N/M)P oxidase. This result prompted us to review the sequence-based pyridoxamine phosphate oxidase annotation for these two unknown ORFs. We found that the protein domain associated with P(N/M)P oxidase GO is actually present in a wide range of proteins (over 39000) that have a plethora of substrates and activities.

We propose that the presence of at least two Pfam domains associated with experimentally confirmed pyridoxamine phosphate oxidases would be a better predictor for this specific function and that *YLR456W*, *YPR172W* and, most probably, all of its homologs have a different functional role. This work calls attention to the limitations of computationally based annotation and the importance of experimental validation for the determination of molecular function, especially for proteins belonging to a widespread and diversified group such as P(N/M)P oxidases.

## Materials and Methods

### Protein cloning, expression and purification

**Fig 2:**
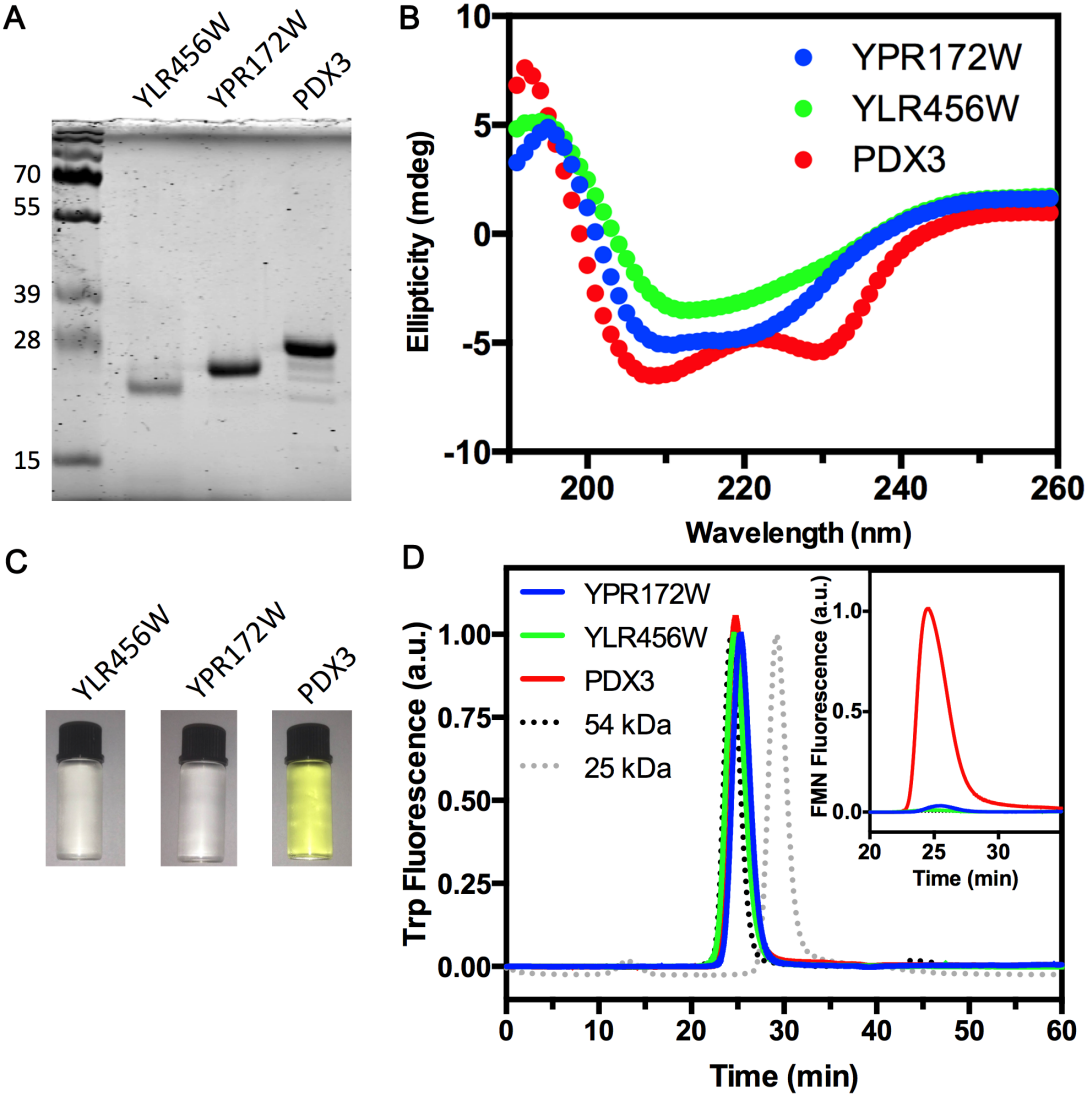
The recombinant Ylr456w, Ypr172w and Pdx3 proteins are folded and form dimers. (A) SDS-PAGE of each purified recombinant protein. (B) Circular dichroism comparing the secondary structure content of the purified proteins. Protein concentration was 40 μM and spectra were generated at 25°C. (C) General aspect of the protein samples at 40 μM. Note that purified Pdx3 presents the characteristic yellow color of proteins that are bound to FMN. Ylr456w and Ypr172w preparations were colorless. (D) Size-exclusion chromatography of 10 μM Ylr456w, Ypr172w and Pdx3 in Superdex 75 3.3/300 column. The injected volume was 10 μL for each sample. The column was calibrated using transthyretin tetramers (54 kDa) and chymotrypsin monomers (25 kDa). The main panel shows tryptophan fluorescence emission at 320 nm (Ex = 280 nm). Inset shows FMN fluorescence emission at 520 nm (Ex = 450 nm). Ypr172 data are shown in blue; Ylr456w in green and Pdx3 in red. All experiments were repeated at least twice with different proteins batches with similar results. A representative result of each experiment is shown.

*YLR456W* ORF was amplified from *Saccharomyces cerevisiae* BY4741 strain cDNA by PCR using the following primers pairs: 5’ **CAT ATG** AAA CTC AAT GAA CAA ATA CCA AA 3’and 5’ **GGA TCC** TTA AAC TGG TTG TAC TG 3’containg *Nde*I and *BamH*I restrictions sites (in bold). The PCR product was cloned into the pET28a plasmid (Novagen). *YPR172W* and *PDX3* sequences were obtained from *Saccharomyces* Genome Data Base (strain S288C), synthesized and cloned into pET28a using *Nde*I and *BamH*I restriction sites (GenScript Company). These constructs code for proteins containing an N-terminal His-tag followed by a thrombin digestion site. All constructs were verified by sequencing the cloning sites. Protein expression was performed with *E*. *coli* BL21 DE3 Rosetta strain (Novagen) grown in LB media. When the culture reached OD_600nm_ = 0.5, expression was induced with 1 mM IPTG for 5 hours at 37°C. Cellular lysis was performed in buffer A, 20 mM Tris, 150 mM NaCl, 1 mM DTT pH 7.4 in the presence of a protease inhibitor cocktail (Roche). The lysate was applied to a His Trap HP column (Life-Technologies). The column was washed with buffer A, and the recombinant protein was eluted with buffer A containing 150 mM imidazole. The pooled fraction containing the recombinant protein was incubated with 50 nM thrombin (Sigma-Aldrich) in the presence of 0.5 mM CaCl_2_. during 16 hours at 25°C. The digested sample was further purified by gel filtration using a Superdex 200 10/300 GL column (Life-technologies) equilibrated with buffer A. The protein concentration was estimated from the absorbance at 280 nm using the predicted extinction coefficient at 280 nm (Ypr172w 21430 M^-1^ cm^-1^; Ylr456w 15930 M^-1^ cm^-1^; Pdx3 47440 M^-1^ cm^-1^) and confirmed by the Bicinchoninic acid (BCA) protein quantitation assay (Thermo Scientific). Protein purity and composition were confirmed by mass spectroscopy. The expected molecular weight after thrombin digestion are (22788 Da for Ypr172w; 23574 Da for Ylr456w; and 27189 Da for Pdx3). The transthyretin (54 kDa) protein used as standard in Fig 2D was purified as previously described [14] and the chymotrypsin (25 kDa) was purchased from Sigma.

### Circular dichroism

Samples containing 40 μM protein in 20 mM Tris, 150 mM NaCl, 1 mM DTT pH 7.4 were analyzed by far-UV CD measurements on a JASCO spectropolarimeter with a 0.01 mm path length quartz cuvette. Data were averaged from three scans at a speed of 100 nm/min, collected in 1 nm steps at 25°C. The baseline (buffer alone) was subtracted from the protein-containing spectra.

### PNP synthesis

The PNP synthesis was done as previously described [15]. Briefly, 15 mg (0.4 mmol) of NaBH_4_ was slowly added to a solution containing 100 mg (0.37 mmol) PLP in 10 mL MeOH. After 30 minutes, the dark yellow solution became light yellow. A few drops of acetone were added and the solution was incubated for 30 minutes to allow the complete consumption of excess NaBH_4_. To dissociate the borate-PNP complex, 5 mL of 10% NaOH was added to the solution and incubated for 10 minutes. The resulting solution was applied to a 1-cm i.d. column containing 10 mL Amberlite-67. The column effluent was applied into a flash column. After volume reduction under negative pressure, a white solid was obtained with 65% yield and 210–212°C melting point.

Structural characterization of the synthesized compound was done by spectroscopic methods, magnetic resonance measurements, and thermal studies. Melting point was determined with a Quimis 340 apparatus and is uncorrected. The purity of the derivative was determined by HPLC using a Shimadzu – LC20AD apparatus equipped with SPD-M20A detector (Diode Array). The column was Kromasil 100-5C18 (4.6 mm/6250 mm). The mobile phase was CH_3_CN:H_2_O (1:1) and 20 μL of sample was injected. The absorbance was measured at 254 nm and the flow was 1 mL/min. NMR analysis was carried out in Varian MR-400 400 MHz spectrometer. ^1^H NMR spectrum was obtained in deuterated chloroform or dimethyl sulfoxide containing 1% tetramethylsilane as an internal standard.

NMR chemical shifts: ^1^H NMR (400 MHz, D_2_O) δ (ppm) 8.2 (1H), 5.0 (2H), 4.9 (2H), 2.6 (3H). % purity = 98.9% by HPLC-C18 (Rt = 2.23 min). Reagents and solvents were purchased from commercial suppliers and used as received. PMP was purchased from Sigma.

### P(N/M)P oxidase activity and FMN binding analysis

The P(N/M)P oxidase activity was measured as previously described [16]. Briefly, five μM Ylr456w, Ypr172w, or Pdx3 were incubated in 0.1 M Tris pH 8.4 containing 10 μM FMN and 1.5 mM PNP. The production of pyridoxal-5'-P was quantified over time by following the increase in absorbance at 414 nm corresponding to the Schiff base formed between pyridoxal-5'-P and Tris. The assay was carried out in 96 well plates at 30°C and absorbance determined by a plate reader spectrophotometer.

The quenching effect of protein binding on FMN fluorescence [17] was explored to compare Ylr456w, Ypr172w and Pdx3 FMN interaction properties. One μM FMN was titrated with increasing concentrations of Ylr456w, Ypr172w or Pdx3. After 2 min incubation at 25°C, FMN fluorescence at 520 nm (Ex = 450 nm) was measured using a spectrofluorometer.

Analytical size-exclusion chromatography was performed using Superdex 75 3.3/300 (Life-technologies) column equilibrated with buffer A. Protein preparations (30 μM) were incubated with 30 μM FMN during 30 minutes. Chromatograms were obtained by injecting samples of 10 μL at a 0.05 mL/min flow rate. Elution was monitored by fluorescence (FMN, Ex = 450 nm, Em = 520 nm and tryptophan, Ex = 280 nm, Em = 320 nm) and absorbance at 280 nm.

### Protein sequence comparison and search for *Ypr172w* and *Ylr456w* homologs

Protein alignment was generated by Clustal-W2 [18] and analyzed using Jalview 2.6.1 [19]. The non-redundant database of protein sequences at the National Center for Biotechnology Information (NCBI) was searched using the PSI-BLAST program with the Blosum62 matrix and other default parameters [20,21]. Each search resulted in a list of homologs, which was added to the next round of PSI-BLAST iteration searches, and each search continued until no new sequences with an alignment score above the default threshold were retrieved. The same methodology was followed for DELTA-BLAST [22] searches. The sequence logo was created using the program Weblogo 3 [23].

All information about Pfam and Interpro protein domains PF01243 and PF10590 was retrieved from Pfam database version 28 at the website http://pfam.xfam.org [24]. Information about each protein structural model associated to these two domains was retrieved from Protein Data Bank (PDB) website (http://www.rcsb.org/pdb/). Protein structure graphics were generated using UCSF-Chimera [25]. The Ven diagram was generated by the eulerAPE software [26].

## Results and Discussion

### Biochemical characterization of *S*. *cerevisiae* Pdx3 and the two putative P(N/M)P oxidases Ylr456w and Ypr172w

*Saccharomyces cerevisiae PDX3* and the two other ORFs coding for putative pyridoxamine phosphate oxidase *YLR456W* and *YPR172W* were heterologously expressed in *E*. *coli* and purified by affinity chromatography. All proteins were soluble and ran as single-band monomers of approximately 25 kDa in a SDS-PAGE (Fig 2A). Pdx3 (expected molecular mass of 27.2 kDa) presented lower mobility compared to Ypr172w (22.7 kDa) and Ylr456w (23.6 kDa). However, Ylr456w presented greater mobility than Ypr172w, which was unexpected considering the differences in molecular mass between these proteins. Nevertheless, the predicted molecular mass for all proteins was confirmed by MALDI-TOF mass spectrometry (data not shown), excluding the possibility of protein degradation.

The secondary structure was investigated by circular dichroism (Fig 2B). All samples generated spectra with characteristics of a well-folded protein containing both beta sheet and alpha helix secondary structure elements. Pdx3 spectrum was quite similar to the one reported for pig P(N/M)P oxidase [27], with characteristic absorption bands at 208 and 230 nm. The two unknown proteins spectra differed considerably from Pdx3 spectrum. This result suggests that Ypr172w and Ylr456w may share a common fold that is different from a typical P(N/M)P oxidase.

The next step involved the characterization of the oligomeric state of the proteins. First, it is important to note that other P(N/M)P oxidases heterologously expressed in *E*. *coli* are usually purified bound to FMN and show a distinctive yellow color [15,28]. This feature was observed with our Pdx3, but not with Ylr456w or Ypr172w preparations (Fig 2C). The three proteins were analyzed by analytical size exclusion chromatography (Fig 2D). All proteins eluted with retention times around 25 minutes as observed with a 54 kDa tetramer of transthyretin [14]. This indicates that the putative P(N/M)P oxidases and Pdx3 are migrating as dimers, a common feature of pyridoxamine phosphate oxidases. When the FMN intrinsic fluorescence (Ex = 450 nm, Em = 520 nm) was used to plot the chromatogram, only Pdx3 produced a single peak at the same retention time observed with tryptophan fluorescence (Fig 2D, inset, red trace). This result corroborates that only Pdx3 is associated to FMN after purification.

**Fig 3:**
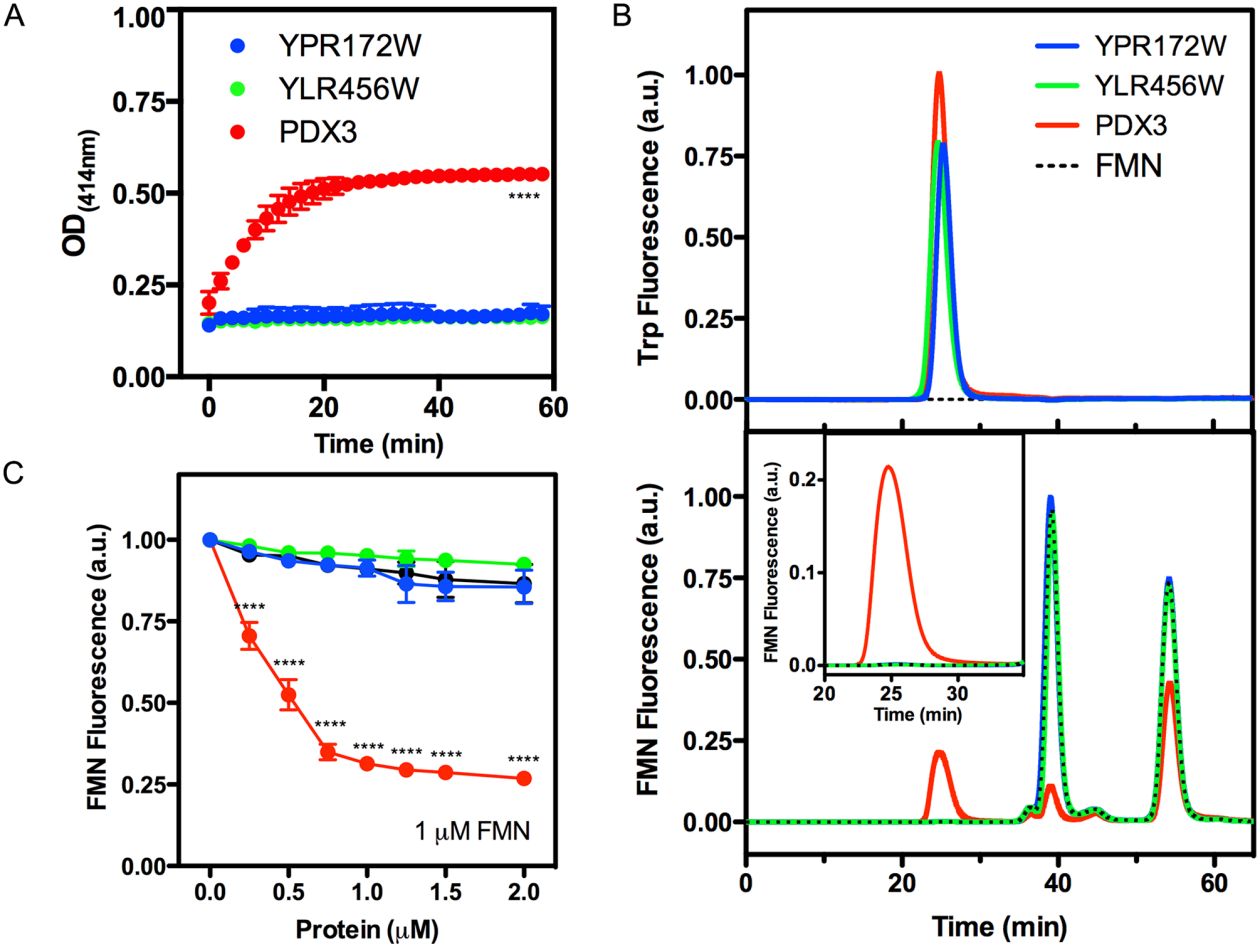
Ylr456w and Ypr172w proteins do not exhibit PNP oxidase activity and cannot bind FMN. (A) PNP oxidase activity test. Five μM Ylr456w, Ypr172w or Pdx3 was incubated with 10 μM FMN and 1.5 mM PNP. The production of pyridoxal 5'-phosphate over time was quantified by following the increase in absorbance at 414 nm, corresponding to the Schiff base formed between pyridoxal-5'-P and Tris present in the buffer. (B) FMN protein binding analysis. Size-exclusion chromatograms of Ylr456w, Ypr172w and Pdx3 in Superdex 75 3.3/300 column. Proteins at 30 μM concentration were incubated with 30 μM FMN at room temperature for 30 min before SEC. The injected volume was 10 μL for each sample. The upper panel shows the chromatogram monitored by tryptophan fluorescence at 320 nm (Ex = 280 nm). The bottom panel shows the fluorescence emission of FMN at 520 nm (Ex = 450 nm). A higher magnification of this chromatogram on the protein dimer elution time can be seen in the inset. Note the absence of FMN fluorescence in Ylr456w and Ypr172w chromatograms (blue and green traces compared to red traces). (C) The quenching effect of protein binding on FMN fluorescence at 520 nm (Ex = 450 nm) was measured on a spectrofluorometer. One μM FMN was titrated with increasing Ylr456w, Ypr172w or Pdx3 concentration. The black circle represents the effect of FMN dilution over the titration curve. All experiments were repeated at least twice with different proteins batches and we show representative chromatograms. Error bars represent the standard deviation, and statistical test used was 2way ANOVA Dunnett’s multiple comparison test where **** represents p<0.0001. In panel A, Pdx3 was statistically different (p<0.0001) compared with Ypr172w and Ylr456w in every point of the kinetics from 2 min to 60 min.

The next step was to determine whether the proteins had P(N/M)P oxidase activity. The activity was determined in the presence of FMN and the substrate pyridoxine phosphate (PNP). The product of the reaction is pyridoxal phosphate (PLP), an aldehyde that reacts with free amines in the reaction buffer forming a Schiff base that can be detected by its absorbance at 414 nm [16]. As expected, the Pdx3 preparation readily catalyzed PLP formation, but we failed to observe PLP synthesis in the presence of Ylr456w or Ypr172w (Fig 3A). We assayed the unknown proteins over a wide range of FMN and PNP concentrations with no activity detected. Also, the alternative substrate PMP was tested with Ylr456w but no reaction took place (data not shown).

Even though Ylr456w and Ypr172w do not react with PNP (Fig 3A), they may still be FMN binding proteins. We investigated this possibility by two different approaches. First we repeated the analytical gel filtration as shown in Fig 2D, but this time, with samples that were incubated with FMN at 1:1 molar ratio. However, no FMN co-eluted with Ylr456w or Ypr172w protein peaks (25 min retention time). We just observed FMN fluorescence at 40 min and at 55 minutes, which corresponds to free FMN, without protein association (Fig 3B, lower panel—compare with the doted lines corresponding to FMN alone). On the other hand, Pdx3 showed FMN binding (Fig 3B, lower panel red trace, detail in inset) and the reduction of free FMN signal in comparison to Ylr456w and Ypr172w chromatograms (Fig 3B, compare red trace with blue, green, and doted traces at 40 and 55 min retention time), suggesting that the purified Pdx3 was not saturated and could still bind FMN (Fig 3B). The same experiment was repeated with FMN:protein at 3:1 molar ratio but similar result was obtained (data not shown).

Another way to detect FMN binding is by measuring the fluorescence quenching caused by protein interactions. For this experiment 1 μM FMN was titrated with increasing protein concentrations and the fluorescence measured on a spectrofluorometer. Pdx3 produced a clear quenching effect due to FMN binding, but Ylr456w and Ypr172w did not (Fig 3C). Moreover, the presence of the putative substrates and products PNP and PLP had no effect on FMN binding (S1 Fig). Finally, we also tested if the addition of Ylr456w or Ypr172w in the reaction buffer at equimolar concentration would interfere with Pdx3 activity, indicating protein interaction or substrate competition. But no differences were observed (results not shown). Together, these results suggest that Ylr456w and Ypr172w cannot catalyze PNP oxidation or bind FMN.

### Primary sequence comparisons between Ylr456w/ Ypr172w and other P(N/M)P oxidases

Since Ylr456w and Ypr172w did not show the expected enzymatic activity, we undertook a careful sequence comparison between Ylr456w and Ypr172w and other characterized P(N/M)P oxidases, aiming to define a better predictor of pyridoxamine phosphate oxidase activity based on the primary sequence.

**Fig 4:**
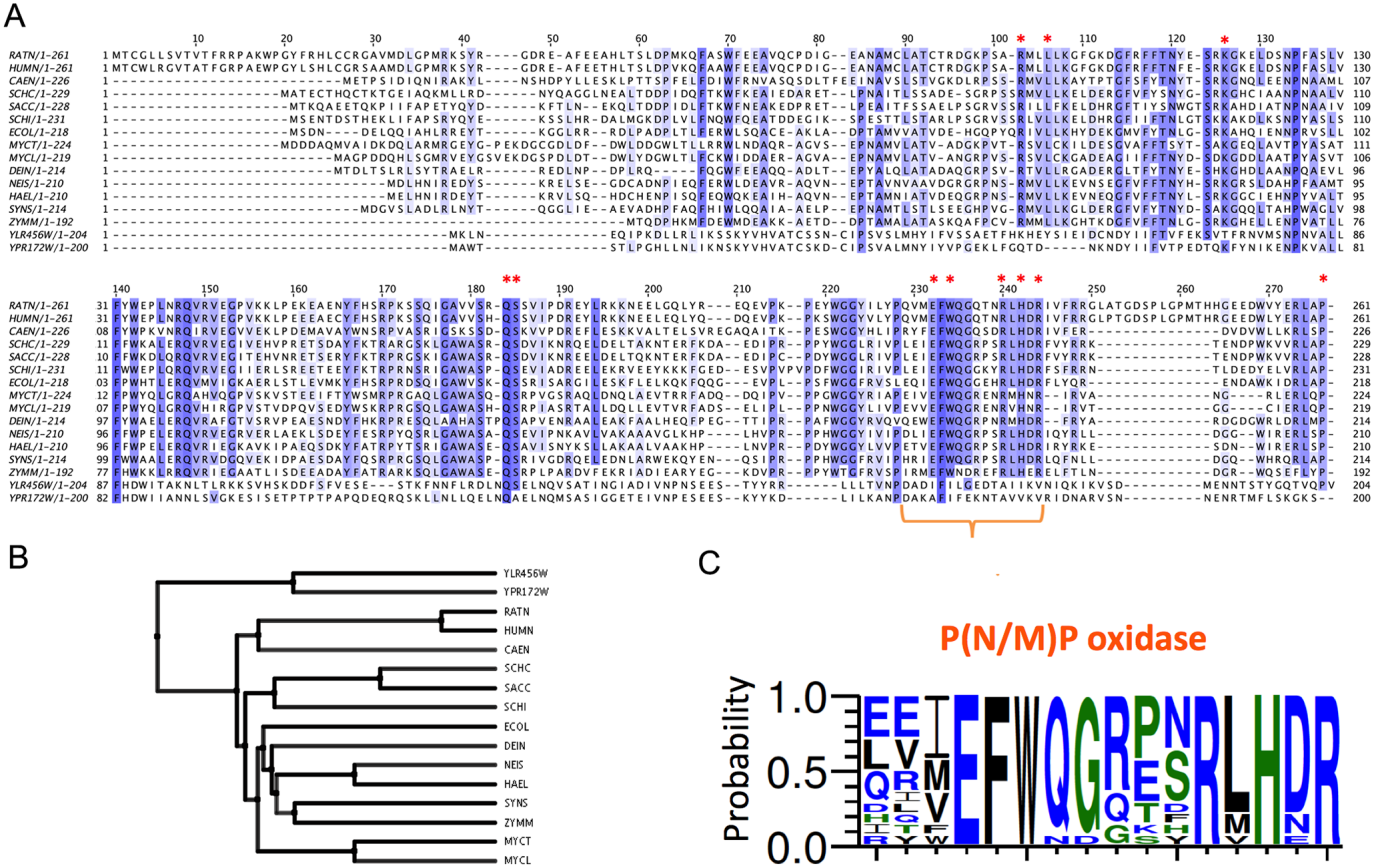
Multiple sequence alignment of P(N/M)P oxidases and putative P(N/M)P oxidases. (A) Protein alignment of putative P(N/M)P oxidases from *S*. *cerevisiae YLR456W* (AAS56557), *YPR172W* (NP_015498) and characterized P(N/M)P oxidases: RATN, *Rattus norvegicus* (O88794); HUMN, *Homo sapiens* (AK001397); CAEN, *Caenorhabditis elegans* (AAA21167); SCHC, *Schizophyllum commune* (AAC28862); SACC, *Saccharomyces cerevisiae* (P38075); SCHI, *Schizosaccharomyces pombe* (CAB60247); ECOL, *Echerichia coli* (P28225); MYCT, *Mycobacterium tuberculosis* (O06207); MYCL, *Mycobacterium leprae* (O33065); DEIN, *Deinococcus radiodurans* (AAF10072); NEIS, *Neisseria meningitidis* (CAB84799); HAEL, *Haemophilus influenzae* (P44909); SYNS, *Synechocystis sp*. (P74211); ZYMM, *Zymomonas mobilis* (AAD53919). Protein Alignment was generated by Clustal-W2. Red asterisks indicate residues that are 100% conserved among P(N/M)P oxidases and directly involved in the binding of FMN. (B) Phylogenetic tree representation of the alignment shown in A. This is a neighbor-joining tree without distance corrections. (C) Sequence logo of 50 P(N/M)P oxidases homologous to *PDX3* showing the most conserved residues in P(N/M)P oxidases, charged residues are shown in blue, polar residues in green and non-polar in black. The motif EFWxxxxxRxHxR is delimited by the orange bracket on panel A.

Fig 4A shows a protein sequence alignment for Ylr456w, its homolog Ypr172w, and another 14 P(N/M)P oxidases experimentally characterized from different species of vertebrates, invertebrates and microorganisms. The tridimensional protein structure of the *E*. *coli* P(N/M)P oxidase bound to FMN (pdb 1dml) revealed 11 highly conserved residues that have non-covalent interactions with FMN; they are identified by red asterisks in Fig 4A [12]. Ylr456w and Ypr172w preserved just 3 and 2 similar residues in these positions, respectively, whereas Pdx3, the only confirmed P(N/M)P oxidase from yeast, presented all 11 residues. The two unknown ORFs show important differences in a C-terminal sequence stretch that concentrates many conserved residues involved in FMN biding. Fig 4C shows a weblogo representation of this motif that is present only in characterized P(N/M)P oxidases. These important differences in protein sequence suggest that the Ylr456w and Ypr172w dimers have very different structural/chemical properties from active P(N/M)P oxidases. These differences probably preclude FMN binding, thereby corroborating the experimental result obtained for the Ylr456w and Ypr172w protein. Moreover, a phylogenetic tree representation of this protein alignment shows that Ylr456w and Ypr172w are clustered in a branch separated from the *S*. *cerevisiae* Pdx3 cluster, which contains other biochemically characterized P(N/M)P oxidases (Fig 4B).

In fact, our experimental observations indicating that Ylr456w and Ypr172w do not have P(N/M)P oxidase activity are in agreement with previous results showing that a mutation in *PDX3* was sufficient to induce all of the phenotypes associated with problems in vitamin B6 biosynthesis, which suggests that *S*. *cerevisiae* has no redundant genes for P(N/M)P oxidase [13].

We then analyzed the distribution of Ylr456w homologs across different species. A Position-specific-iterated BLAST (PSI-BLAST) [20] search for Ylr456w homologs returned 477 proteins, 434 of which were derived from fungi and 43 from Actinobacteria. A similar result is observed with Ypr172w. Most sequences had an unknown function or were named as putative P(N/M)P oxidases or putative FMN binding proteins. Ypr172w and Ylr456w share 48% identity and homologs of both proteins are present in *Saccharomyces* species derived after the whole genome duplication event [10].

PSI-BLAST failed to return Pdx3 and other confirmed P(N/M)P oxidases as Ylr456w and Ypr172w homologs. The only search method that included confirmed P(N/M)P oxidases to the search results was enhanced-lookup time-accelerated BLAST (DELTA-BLAST) [22]. Like PSI-BLAST, DELTA-BLAST uses a position-specific score matrix (PSSM) to enhance BLAST sensibility. A PSSM is derived from a multiple sequence alignment of related proteins, and models the amino-acid substitutions particular to a specific protein family and sequence position. PSI-BLAST automatically generates a PSSM from the results obtained after the first iteration of database search. The PSSM assists in the detection of more distantly related homologs in subsequent iterations. DELTA-BLAST uses a similar rationale, but PSSM is derived from pre-constructed sequence alignments used to obtain the conserved domain database (CDD) [29]. CDD is a protein annotation resource that gathers domain models curated by NCBI and imported from other databases (e.g. Pfam). The better quality of the alignments used to construct the CDD protein families, which take into account secondary and tertiary protein structure information, is one of the factors accounting for the enhanced sensitivity of DELTA-BLAST [22].

**Fig 5:**
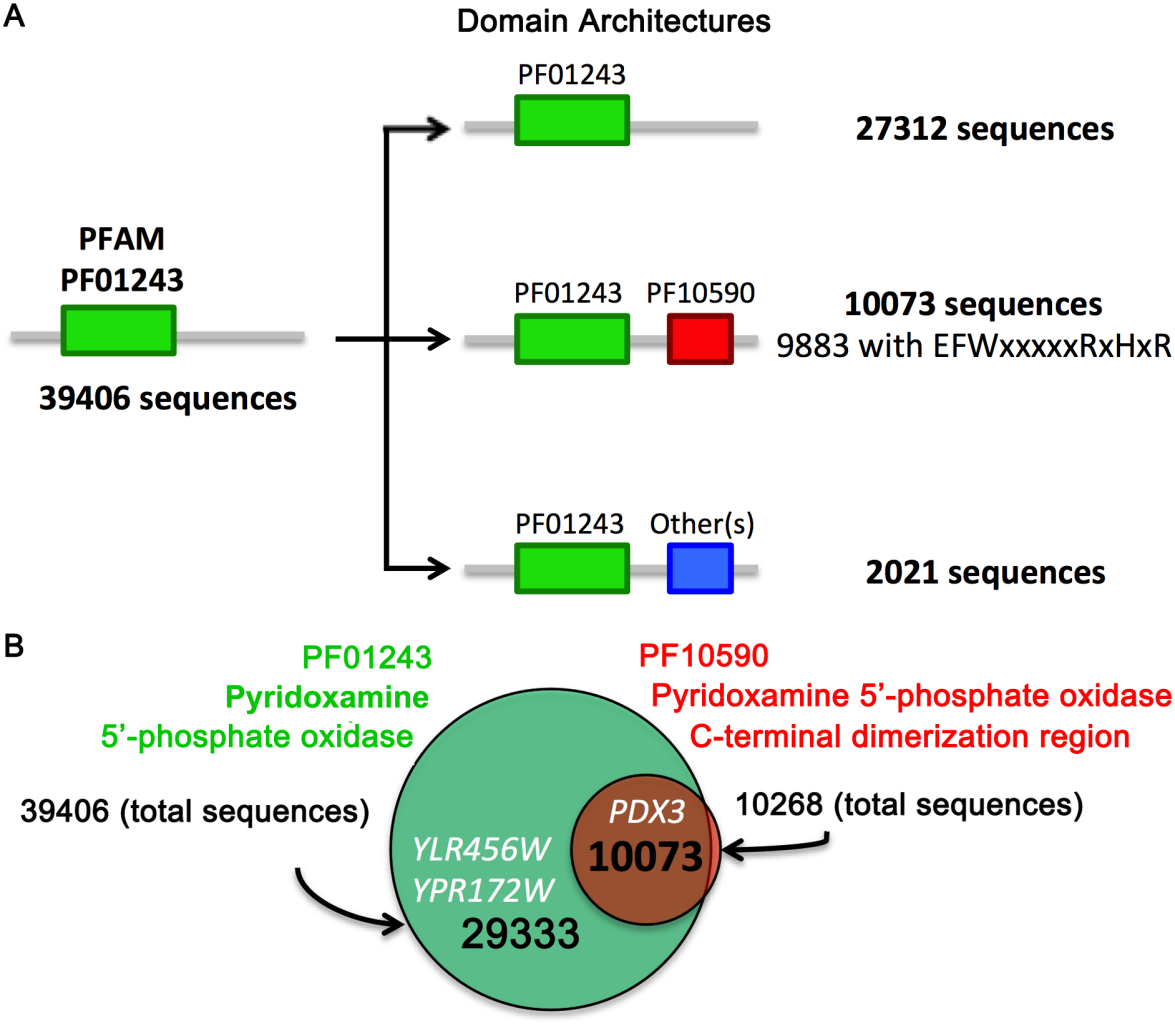
Domain organization of Pfam family PF01243. (A) Diagram showing domains associated to Pfam P(N/M)P oxidase protein family. PF01243 comprises 39406 sequences; 27312 have only the Pyridox_oxidase domain, and none of these sequences possess the motif EFWxxxxxRxHxR. In contrast, 10073 sequences also possess the Pyridox_oxidase C-terminal dimerization domain PF10590, and 98.1% of these sequences possess the motif EFWxxxxxRxHxR. (B) A Venn diagram showing the number of proteins presenting P(N/M)P oxidase domains. 39406 sequences have the domain PF01243 (in green). The domain PF10590 is present in 10268 sequences (in red) and 10073 sequences have both domains. *YLR456W* and its homolog *YPR172W* have only the PF01243 domain, while *PDX3* has both.

By the same token, SGD automatic annotation is based on InterPro and Pfam protein families database. Ylr456w, Ypr172w and their homologs were classified as putative P(N/M)P oxidases based on an N-terminal segment (9–92 and 8–86 respectively) that was detected by the profile hidden Markov model (HMM), which defines the Pfam family PF01243 Pyridox_oxidase (Fig 5). According to the current edition of Pfam (28.0), PF01243 includes 39406 sequences [24]. It is important to note that approximately 30% of the sequences that contain PF01243 also contain additional Pfam domains, thereby forming 64 different architectures, or domain combinations (Fig 5A).

We observed that all of the experimentally confirmed P(N/M)P oxidases in Fig 4A contain two specific domains associated with Pyridoxamine oxidase function: the aforementioned N-terminal PF01243 Pyridox_oxidase domain and the C-terminal domain, PF10590, which is not present in the Ylr456w and Ypr172w sequences (Fig 5). Almost all of the proteins containing PF10590 also contain the N terminal domain PF01243, with a few exceptions that are not experimentally characterized (Fig 5B). Further, though PF10590 is named as a pyridoxamine 5'-phosphate oxidase C-terminal dimerization region (PNPOx_C), the HMM defining this domain includes many residues that are involved in FMN binding, including the almost invariable EFWxxxxxRxHxR stretch present in all confirmed pyridoxamine 5'-phosphate oxidases (Fig 5).

This observation raised the hypothesis that the presence of PF01243 and PF10590 might be a more reliable indicator of pyridoxine 5'-phosphate oxidase function than PF01243 alone. We analyzed the tridimensional structures associated with the proteins belonging to these families and searched for direct and indirect evidence of pyridoxine 5'-phosphate oxidase activity, such as publications analyzing the protein function [30–44] or simply the presence of FMN or other ligands associated to the tridimensional structure deposited in the RCSB protein data bank (http://www.rcsb.org/pdb/home/home.do). As observed in Table 1, more than half of the structures related only to domain PF01243 showed no evidence of FMN binding in their tridimensional structure. Other structures containing only PF01243 were predicted to have different functions such as lectin (pdb entry: 1AXY), heme oxigenases (pdb entry: 3gas, 3swj, 1vl7), F420-dependent reductases (pdb entry: 3f7e) or FAD binding (pdb entry: 3ec6). The archetypal P(N/M)P oxidases all contain PF01243 and PF10590 domains, but it should be noted that the conservation of the residues involved in substrate binding is important for functionality. A human variant associated with neonatal epileptic encephalopathy disorder (pdb: 3HY8) has a substitution in the second arginine residue of the motif EFWxxxxxRxHxR, resulting in a severe loss of the catalytic activity (catalytic efficiency decrease (*k*cat/K*m*) of 470 fold in comparison to the WT) [17]. Conserving the residues in the N-terminal region is also important, as shown by putative P(N/M)P oxidases from *P*. *aeruginosa* and *P*. *fluorescens* (pdb 1T9M and 1TY9) that can bind FMN but not pyridoxine due to steric hindrance [39].

**Table 1:** List of structures associated to the Pyridox_oxidase domain (PF01243).

Uniprot entry	PDB ID	Pfam 01243	Pfam 10590	Substrate binding^a^	Molecular function^b^	Ref
A0QXP8_MYCS2	3F7E	yes	no	no	unknown	NA
A9CI98_AGRT5	3DNH	yes	no	no	unknown	NA
B9WVW9_STRSU	2HHZ	yes	no	no	unknown	NA
C0LU01_HELPX	3GAS	yes	no	Heme	Heme storage	[30]
A5F0R4_VIBC3	3TGV	yes	no	Heme	Heme storage	[30]
FMNB_DESVM	1AXJ	yes	no	FMN	Not P(N/M)P oxidase, unknown.	[31]
	1FLM					[32]
	1WLI, 1WLK, 3A20					[33]
	2E83, 3AME, 3AWH					NA
	3A6Q, 3A6R					[34]
O53240_MYCTU	1RFE	yes	no	no	unknown	NA
Q1GBW8_LACDA	2HTD	yes	no	no	unknown	NA
Q4FV99_PSYA2	2RE7	yes	no	no	unknown	NA
Q5HSH8_CAMJR	3SWJ	yes	no	Heme	unknown	NA
Q81Z55_BACAN	3EC6	yes	no	FAD	unknown	NA
Q825J7_STRAW	2IAB	yes	no	no	unknown	NA
Q8A7U5_BACTN	2FHQ	yes	no	no	unknown	NA
Q8YMA7_NOSS1	1VL7	yes	no	no	unknown	NA
Q8YS45_NOSS1	2I02	yes	no	FMN	unknown	NA
Q926Z8_LISIN	3DB0	yes	no	no	unknown	NA
Q97DI6_CLOAB	2HQ7	yes	no	no	unknown	NA
Q97G05_CLOAB	2IG6	yes	no	FMN	unknown	NA
Q9HW16_PSEAE	2ARZ	yes	no	no	unknown	NA
Y2074_MYCTU	2ASF	yes	no	no	unknown	[35]
Y1155_MYCTU	1W9A	yes	no	no FMN binding	not P(N/M)P oxidase, unknown.	[36]
	1XXO					NA
	1Y30, 2AQ6			FMN/not PNP	unknown	[37]
	4QVB			F420	F420 storage	[38]
O69755_PSEAI	1T9M	yes	yes	FMN, not PNP	Unknown, Phenazine biosynthesis	[39]
PHZG_PSEFL	1TY9	yes	yes	FMN, not PNP	Not P(N/M)P oxidase.	[39]
	4HMS, 4HMT, 4HMU,4HMV			FMN, hexahydrophenazine-1,6-dicarboxylate; tetrahydrophenazine-1-carboxylate	Phenazine biosynthesis	[40]
Q396C5_BURS3	4HMW,4HMX	yes	yes	FMN,hexahydrophenazine-1,6-dicarboxylate; tetrahydrophenazine-1-carboxylate	Phenazine biosynthesis	[40]
PDXH_MYCTU	2A2J	yes	yes	FMN/PNP	PNP oxidase	[15,41]
PNPO_HUMAN	1NRG	yes	yes	FMN and PNP	P(N/M)P oxidase.	[28]
	3HY8					[17]
PDX3_YEAST	1CI0	yes	yes	FMN and PNP	P(N/M)P oxidase.	[13]
PDXH_ECOLI	1DNL	yes	yes	FMN and PNP.	P(N/M)P oxidase.	[12]
	1G76, 1G77, 1G78,1G79					[42]
	1JNW					[43]
	1WV4					[44]

^a^ Molecules in the deposited structure or experimentally tested for biding;

^b^ Experimentally validated.

In conclusion, our experimental data failed to support the predicted P(N/M)P oxidase function of *YLR456W* and *YPR172W*, indicating that these ORFs and likely all related homologs are involved in different roles. In fact, our analysis revealed that the domains associated with this molecular function, particularly PF01243, have very low predictive power. Proteins that belong to this family seem to adopt diverse functions via slight modifications of the P(N/M)P oxidase protein sequence and fold.

## Supporting Information

S1 Fig Ylr456w and Ypr172w proteins don’t bind FMN. FMN fluorescence (1 μM) under Ylr456w, Ypr172w and PDX3 titration after incubation at 25°C for 2 min in buffer A in the presence of 100 μM PNP (A) or 100 μM PLP (B). Ex = 450 nm, Em = 520 nm. The error bars represent the standard deviation of two independent experiments with two different proteins batches. Statistical test used was 2way ANOVA Dunnett’s multiple comparison test where *, p<0.05; **, p<0.01; ***, p<0.001 and ****, p<0.0001.
(TIFF)
