# Supplementary figures and images for: Experimental Evidence for a Revision in the Annotation of Putative Pyridoxamine 5'-Phosphate Oxidases P(N/M)P from Fungi
Source: PLoS One. 2015 Sep 1;10(9):e0136761. doi: 10.1371/journal.pone.0136761 (PMC4556617; doi:10.1371/journal.pone.0136761)

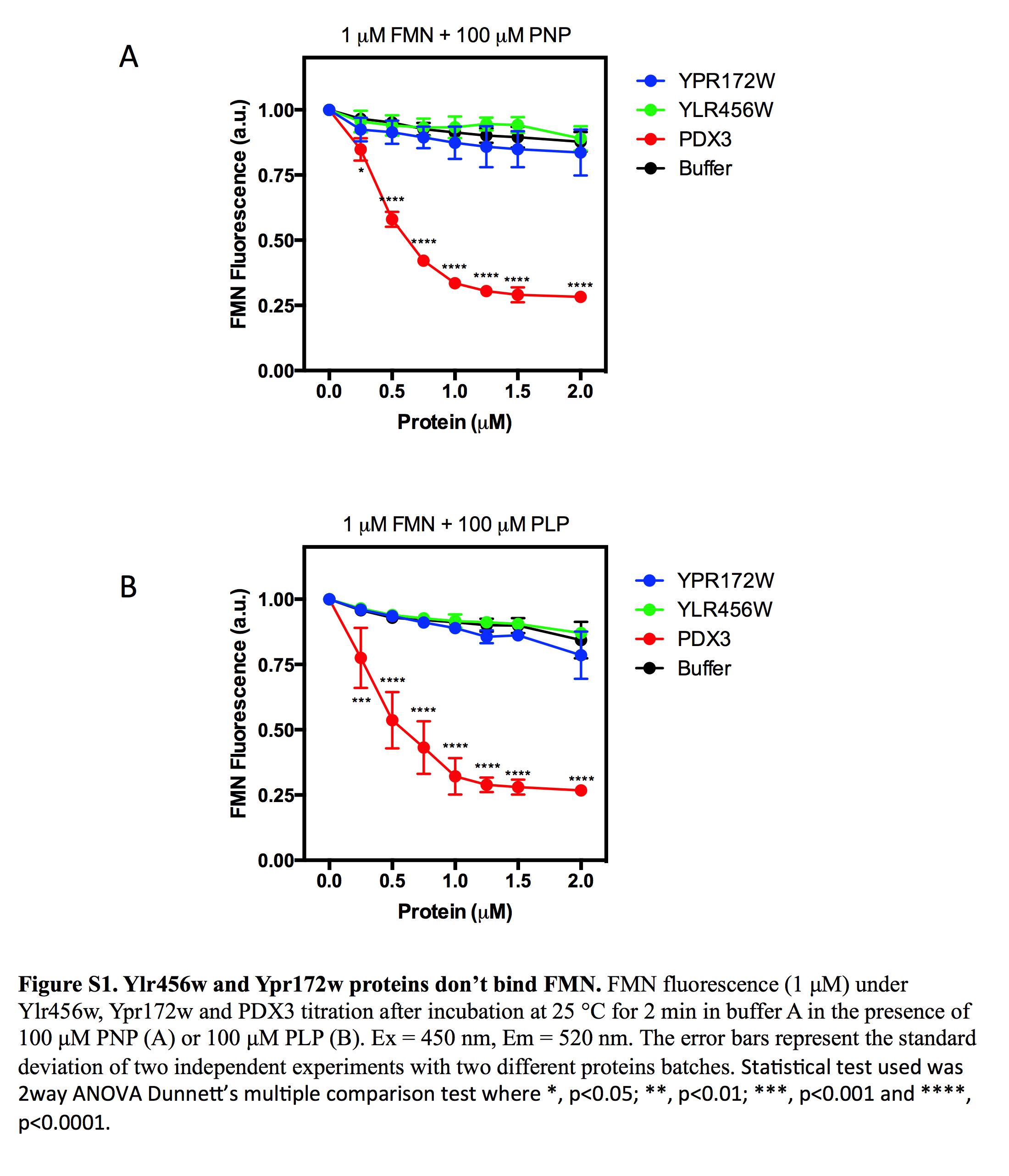

Supplement: S1 Fig — FMN fluorescence (1 μM) under Ylr456w, Ypr172w and PDX3 titration after incubation at 25°C for 2 min in buffer A in the presence of 100 μM PNP (A) or 100 μM PLP (B). Ex = 450 nm, Em = 520 nm. The error bars represent the standard deviation of two independent experiments with two different proteins batches. Statistical test used was 2way ANOVA Dunnett’s multiple comparison test where *, p<0.05; **, p<0.01; ***, p<0.001 and ****, p<0.0001. (TIFF) [file pone.0136761.s001.tiff]
